# New Methods of Treatment in Phthisis

**Published:** 1891-10-31

**Authors:** 


### The Practitioner's Mirror and Retrospect.

[*The Editor will be glad to receive offers of co-operation and contributions from members of the profession. All letters should be addressed to* The Editor, The Lodge, Porchester Square, London, W.]

### MEDICINE.

### NEW METHODS OF TREATMENT IN PHTHISIS.

V.—Loebinger's Insufflations in Phthisis.

In a recent communication* Dr. Hugo Loebinger advocates a method of reaching the tubercular lesions in the lung tissue by means of insufflations of a compound powder, whose basis for the purpose of mechanical action in the diseased tissues is phospide of calcium.

This method is intended for the local treatment of phthisis and is to be associated with all the accessory general treatment which can be brought to bear with advantage on the case. We certainly should never welcome any system or method which was not associated with all the favourable accessories that it is possible to bring to the aid of the patient; certainly it is most undesirable to countenance the unfair risks to which many patients are exposed, while each new method is being tried on its own merits alone. It will be time enough to walk without crutches and rely on one "system" unaided when a method has been devised which, with all additional assistance at our disposal, can maintain its position as a "cure" for phthisis.

"How is it possible to act with purely local effect upon the actual seat of the disease in the lungs?" asks Dr. Loebinger, and he proceeds to answer the question. The primary requirement is the acknowledged one of first locating the seat of the disease, then to circumvent the same after a thorough physical examination, not resting content, in consequence of the discovery of the bacilli found in the sputum, with the general diagnosis—tuberculosis pulmonalis. Proceed then to ascertain the form in which medicinal substances may be conducted direct to the diseased portion of the lung tissue and there remain, in order to discriminate between the inhaled gases (whose contact with the seat of disease must of necessity be transient) deposited for a certain length of time, so that not merely a transitory effect will have been attained. And this is in the form of powder! Can powder (or dust) be conveyed to the lungs? The dust of rooms and streets, daily inhaled, only in part remains in the superior air passages, from there to be again expectorated; a portion penetrates into the lung-parenchyma, where, at autopsies, it is often met with, representing a portion of the pigment of the lung. The occurrences of anthracosis, pneumonokosis, and siderosis in persons following certain vocations are instances in point. It is remarkable that here the inhaled dust, provided, of course, that the same is free from all infectious admixture—following a purely mechanical path will cause, first slight, then more aggravated lesions in the lungs, and being in the form of so-called fibrous induration, occasion cicatrical formations in the interstices of the tissue (viz., interalveolar, interbronchial, and subpleural), with consequent shrinkage. That which is here characterised as a pathological phenomenon is the art of natural cure which seeks to eliminate and make innocuous the destructive micro-organisms by means of reconstruction of the connective tissue of the lungs, producing an actual cicatricial formation, taking the place of the decayed lung parenchyma, which often occurs spontaneously in pulmonary phthisis. Wherefore has Dr. Loebinger thought it well to work upon this plan which is indicated, as it were, by nature.

The insufflated calcium phosphide powder, which becomes an amorphous powder insoluble in water, which, when deposited in the lung tissue, is imbibed by the lymph cells, which in turn become migratory amœboid cells, carrying the powder through the interstitial tissue and finally gaining a foothold in the filter apparatus of the bronchial lymphatic glands. A portion of the calcium phosphide, it is believed, mingling with the albumen of the necrosed tissues, which possesses everywhere in the body a well-known chemical affinity for calcium, becomes chemically united with it, representing calcined lime in the cheesy portions.

To satisfy the demands of antisepsis, which, without first seeking a specific against the bacillus tuberculosis, promises success in view of the fact that phthisis of the lungs, being a mixed process, where other pathogenic bacilli also come into play, sodium benzoate may be mentioned as a second constituent for the powder mixture. As the latter is soluble in water, the effect is not a mechanical but a chemical one, and one that possesses great antizymic strength.

Our third and most significant constituent is either one of the ethereal oils, in the shape of elæosaccharum, so that the whole may form a fine, amorphous powder; for the consistence is lost if the oleaginous constituents rise above ten per cent. They stand at the head of specific contratu-berculous substances, according to Koch and others.

As soon as the powdered mixture is deposited the intermixed ethereal oils pass away, in the form of vapour, from the particles to which they cling, and enter the neighbouring diseased tissues. This is an important fact, for, if cavities do not exist which communicate with the bronchi, then in the most favourable case the powder will reach only to the vicinity of the *locus affectus*, which generally lies apart or excluded, and not directly accessible by way of the respiratory passages. The transpiration of the internal evaporation of oils, for which the less important powder admixtures represent the vehicle only, is termed by Loebinger "secondary internal inhalation."

The following difficulties in applying the method have to be encountered. The first step is to pass the narrow passage of the glottis. This becomes enlarged to its utmost on deep inspiration. Instruct the patient to take a deep breath while the tongue is stretched forth, so as to raise the epiglottis. A thin tube is then inserted, with the end bent at a right angle, and the end is then passed over the open glottis. In order that the propelling force may be sufficiently strong to carry the powder well over the bronchi, Loebinger has had a special spray producer made, the Livingstone spray producer, with the assistance of which a power of from fifty to to sixty pounds to the cubic inch is developed. In order that the powder may pass down the bronchi in a straight line the patient must assume a particular position. Thus, if the disease be located on the left side, the position assumed is as follows: Body bent forward to the right with head thrown back. Great care is needed to be sure that the powder reaches the right spot.

The doctor has followed this method for eighteen months. What are the results? He can affirm that of the numerous cases of pronounced pulmonary phthisis which have been subjected to this treatment, there have been but few negative cases to record, and these but apparently negative, as the treatment was not continued long enough.

Where treatment was possible (and it is never required for a longer period than three or four months), improvement was observable until the disappearance of the bacilli, except in one case; and gradually the cough and expectoration ceased, and an increase of flesh was apparent. After that it was possible to undertake the physical treatment or cure of the

* *New York Medical Journal*, December 20, 1890.

diseased portions within which the desired cicatricial formation had taken place.

Dr. Loebinger gives details of two cases, which seem to promise excellent results from this method, and promises further details of many cases at an early date.

